# Cerebral arterial air embolism secondary to iatrogenic left atrial-esophageal fistula: a case report

**DOI:** 10.1186/s12883-020-1602-1

**Published:** 2020-01-11

**Authors:** Ping Zhang, Yi Bian

**Affiliations:** 1 Department of Neurology, Tongji Hospital, Tongji Medical College, Huazhong University of Science and Technology, Wuhan, China; 2 Department of Emergency, Tongji Hospital, Tongji Medical College, Huazhong University of Science and Technology, No.1095 Jiefang Road, Wuhan, 430030 China

**Keywords:** Cerebral arterial air embolism, Atrial-esophageal fistula, Iatrogenic, Atrial fibrillation ablation

## Abstract

**Background:**

Cerebral arterial air embolism is a life-threatening complication that can result in neurologic deficits or death. Sometimes it is iatrogenic, presented as a complication of invasive medical procedures. Here we describe a case of cerebral arterial air embolism secondary to iatrogenic left atrial-esophageal fistula, of which the diagnosis might be covered up by the complicated pathophysiologic changes.

**Case presentation:**

A 68-year-old man presented with unconsciousness hours after aphasia and right hemiplegia, accompanied with hematemesis and fever. He had a history of atrial fibrillation, treated by radiofrequency catheter ablation 1 month ago. Brain CT displayed massive air embolism in left hemisphere, as well as right parietal lobe. Chest CT demonstrated a focus of air in the left atrium, which highly suggested an atrial-esophageal fistula. The patient received high flow (6 L/min) oxygen therapy. Intravenous antibiotics including imipenem and vancomycin were administered together with crystalloid rehydration. Supportive therapies were given including intubation, mechanical ventilation and vasopressor use. Because of the patient’s unstable condition and poor prognosis, surgical repair was considered but not pursued. The patient presented a very fast deterioration of cardiac function and circulatory failure, and finally died from cardiac arrest.

**Conclusions:**

Clinicians must have a high index of suspicion for atrial-esophageal fistula for patients presenting with chest discomfort, new onset of stroke, upper gastrointestinal bleeding, and development of sepsis as long as 50 days after the ablation for atrial fibrillation. Urgent CT can ultimately establish the diagnosis in most cases.

## Background

Cerebral vascular air embolism occurs when air enters a patient’s cerebral circulation, which may occur either in artery or in vein. It most commonly results from arterial air embolism [1]. Cerebral arterial air embolism is a life-threatening complication that can result in neurologic deficits or death, depending on the volume and scope of gas entry into the cerebral circulation. Sometimes it is iatrogenic, presented as a complication of invasive medical procedures, mechanical ventilation, or anesthesia [1, 2]. The diagnosis is mainly based on the neurologic symptoms and the performance history of an invasive procedure. Here we describe a patient who suffered from cerebral arterial air embolism secondary to iatrogenic left atrial-esophageal fistula, of whom the diagnosis might be covered up by the complicated pathophysiologic changes.

## Case presentation

A 68-year-old man was admitted to the emergency room due to hematemesis and fever, accompanied with palpitation and chest distress. He spit blood twice, with the total volume of 500 ml. His body temperature was 39.5 °C, presented with chills. Twenty hours later, he presented with new symptoms of aphasia and right hemiplegia. And very soon he became unconscious. He had a history of atrial fibrillation, treated by radiofrequency catheter ablation 1 month ago. However after the interventional therapy, he still felt palpitation intermittently.

**Fig. 1:**
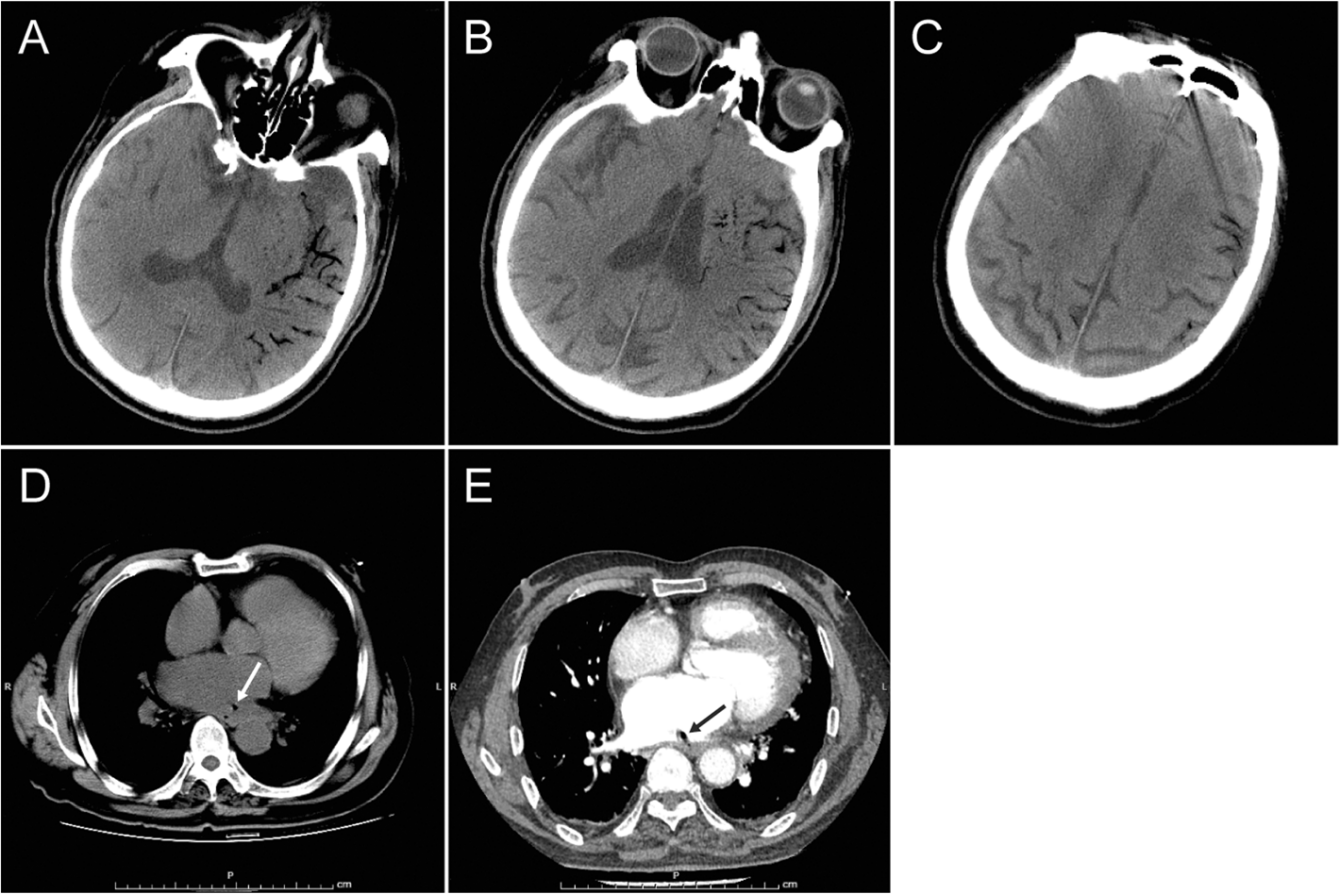
CT scanning. Brain CT shows massive air embolism in the left frontal, parietal, temporal, and occipital lobe (**a**, **b**, **c**), the left basal ganglion (**b**), as well as the right parietal lobe (**c**). Chest CT plain scan (**d**) and contrast-enhanced scan (**e**) show the enlargement of the left atrium, and a focus of air in the left atrium (arrows), which highly suggests an atrial-esophageal fistula

General examination was unremarkable. On neurological examination, he appeared stupor, mixed aphasia and central paralysis of the right side. Blood routine indicated infection and anemia, with leukocyte count 23.23*10^9^/L, neutrophil count 21.59*10^9^/L, hemoglobin 93 g/L, and hematocrit 28%. His C-reactive protein (CRP) level was 140 mg/L, with blood lactate 2.22 mmol/L, serum procalcitonin (PCT) 32.51 ng/mL, which supported the diagnosis of sepsis. His amino-terminal pro-brain natriuretic peptide (NT-proBNP) was 8445 pg/mL, with hypersensitive cardiac troponin I (CTnI) 862.5 pg/mL. Cardiac color ultrasound revealed enlargement of the left atrium (40 mm) and the left ventricle (61 mm), and impairment of the left ventricular systolic function (Ejection Fraction: 30%). Then computer tomography (CT) of the brain was performed, displaying massive air embolism in left hemisphere, as well as right parietal lobe (Fig. 1). Chest CT demonstrated a focus of air in the left atrium (Fig. 1), which highly suggested an atrial-esophageal fistula [3]. The patient received high flow (6 L/min) oxygen therapy since diagnosis. He didn’t receive hyperbaric oxygen therapy because of hemodynamic instability. Intravenous antibiotics including imipenem and vancomycin were administered together with crystalloid rehydration. Supportive therapies were given including intubation, mechanical ventilation and vasopressor use. Because of the patient’s unstable condition and poor prognosis, surgical repair was considered but not pursued. The patient presented a very fast deterioration of cardiac function and circulatory failure, and finally died from cardiac arrest.

## Discussion and Conclusions

Arterial air embolism can be an iatrogenic complication of numerous invasive medical procedures which performed in almost all clinical specialties when an entry of air into the arterial circulation is created [2]. The entry of air can happen through the pulmonary veins or directly into the arteries of the systemic circulation. Among all vulnerable areas, obstruction of either the coronary or the cerebral arteries is especially critical, which sometimes can be fatal [2]. Cerebral arterial air embolism typically involves the small arteries, the average diameter of which ranges from 30 to 60 μm [4]. One the one hand, the reduction in perfusion distal to the obstruction causes hypoxia; on the other hand, the secondary inflammatory response to the air bubble aggravates brain damage [2]. Clinical symptoms of cerebral arterial air embolism can be decreased consciousness, headache, dizziness, seizures and focal neurological defects, which vary with the location and size of the air bubbles [5]. The most important criterion for the diagnosis is the patient’s history, which reveals the temporal relation between the initial neurologic symptoms and the performance of an invasive procedure [2]. CT scan and magnetic resonance imaging (MRI) of the brain can sometimes help making the diagnosis.

**Fig. 2:**
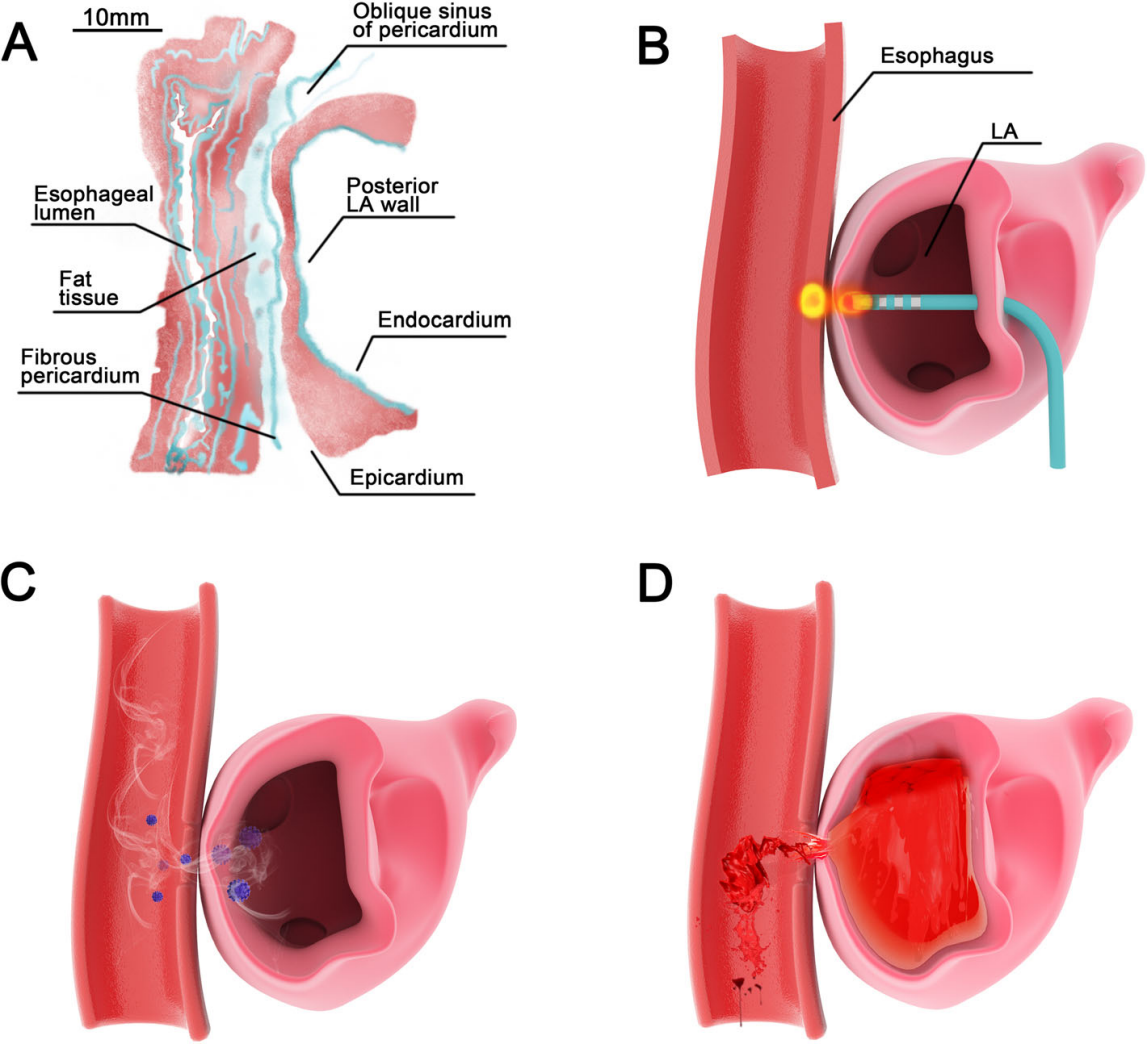
Schematic diagram of atrial-esophageal fistula formation and consequent pathogenetic conditions. **a** Histological section diagram shows the distance between the left atrial wall and the esophageal wall, which can be as small as 5 mm. **b** Thermal injury to the atrial esophageal junction is produced during the ablation procedure. This leads to esophageal injury and atrial-esophageal fistula formation. **c** The air and bacteria in the esophagus can be introduced through the fistula to the arterial circulation. **d** The blood is lost from the left atrium to the esophagus through the fistula

Atrial-esophageal fistula after radiofrequency catheter ablation is a rare but fatal complication. This complication was first described in the surgical literature in 2001 [6] and in the medical literature in 2004 [7]. The incidence is estimated to be less than 0.1 to 0.25% of atrial fibrillation ablation procedures [8]. Its mortality has been reported to be 67 to 100% [9, 10]. Its clinical manifestations mainly include neurologic symptoms, bacteremia and cardiac complications [11]. Neurologic symptoms include encephalopathy, seizures, transient ischemic attack (TIA), septic embolic stroke, and air embolism [3], which can appear alone or in combination. In 2017, Fatula et al. [12] reviewed all reported cases of atrial-esophageal fistula to date (*N* = 75). The authors reported that fevers (76.7%) and neurological deficits (69.9%) were the most common presenting symptoms. Successively, our patient presented almost all common symptoms, including fever, neurological deficits, chest pain, hematemesis, and altered mental status. Esophageal injury during the ablation procedure is the leading cause of the formation of atrial-esophageal fistula. Autopsy studies have demonstrated that the distance between the left atrium and the esophagus can be as small as 5 mm [13]. The ablative damage and perforation of the atrial wall and adjacent esophagus can lead to the development of an abnormal anatomic passage between the left atrium and the esophagus [14]. The time course of the presentation of atrial-esophageal fistula after the ablation procedure has been reported from within a few days to as long as 50 days [15]. In our patient, the prolonged timeframe of 1 month and the nonspecific symptoms at the very beginning might greatly mislead the diagnosis. But ultimately the diagnostic clues were manifested through the air embolism and the consequent neurologic symptoms. The cerebral air embolism, sepsis and “hematemesis” (blood loss from the left atrium to the esophagus through the fistula) all supported the diagnosis of atrial-esophageal fistula. The iatrogenic left atrial-esophageal fistula secondary to atrial fibrillation ablation formed abnormal anatomic communication tract between the esophagus and the circulation. The air was introduced through the fistula to the arterial circulation, resulting in multiple cerebral arterial air embolism. The food and bacteria in the esophagus got into the blood, causing sepsis and fever. The “hematemesis” originated in blood loss from the left atrium to the esophagus through the fistula (Fig. 2). Our patient demonstrated the complexities of establishing the diagnosis. For the diagnosis of atrial-esophageal fistula, the optimal evaluation method is not transthoracic echocardiogram but chest CT [16]. When chest CT shows air in the mediastinum or pericardium, or intracardiac air, an atrial-esophageal fistula is strongly suggested [3]. Transesophageal echocardiography or endoscopy is relatively contraindicated, because of the potential risk of developing air embolism [9].

Considering the high mortality rate and the poor prognosis, neurologists need to be aware of this rare but fatal complication in patients with new neurological deficits and recent history of ablation for atrial fibrillation. Unfortunately, neurological deficits tend to be a late finding, which often result in substantial sequelae and increased mortality. The variability in both timing and clinical presentation often leads to delayed diagnosis of atrial-esophageal fistula. Clinicians must have a high index of suspicion for this diagnosis for patients presenting with chest discomfort, new onset of stroke, upper gastrointestinal bleeding, and development of sepsis as long as 50 days after the ablation. Urgent CT can ultimately establish the diagnosis in almost all patients. Even though mortality is extremely high, earlier recognition and multidisciplinary collaboration (including cardiology, anesthesiology, cardiothoracic surgery, gastroenterology, and neurology) may provide an opportunity for improving outcomes.

## Data Availability

All data generated or analyzed during this study are included in this published article.

## Abbreviations

CRP: C-reactive protein
CT: Computer tomography
CTnI: Cardiac troponin I
MRI: Magnetic resonance imaging
NT-proBNP: Amino-terminal pro-brain natriuretic peptide
PCT: Procalcitonin
TIA: transient ischemic attack
